# Mechanism of syncope: role of ambulatory blood pressure monitoring and cardiovascular autonomic function assessment

**DOI:** 10.1093/eurheartj/ehae907

**Published:** 2024-12-30

**Authors:** Antonella Groppelli, Vincenzo Russo, Erika Parente, Angelo Comune, Frederik J de Lange, Giulia Rivasi, Martina Rafanelli, Jean Claude Deharo, Jaume Francisco-Pascual, Roberto Maggi, Artur Fedorowski, Andrea Ungar, Gianfranco Parati, Michele Brignole

**Affiliations:** Department of Cardiology, IRCCS Istituto Auxologico Italiano, Faint and Fall Research Centre, S. Luca Hospital, Piazzale Brescia 20, Milano 20149, Italy; Department of Translational Medical Sciences, Cardiology and Syncope Unit, University of Campania ‘Luigi Vanvitelli’—Monaldi Hospital, Piazzale E. Ruggeri, Naples 80126, Italy; Department of Translational Medical Sciences, Cardiology and Syncope Unit, University of Campania ‘Luigi Vanvitelli’—Monaldi Hospital, Piazzale E. Ruggeri, Naples 80126, Italy; Department of Translational Medical Sciences, Cardiology and Syncope Unit, University of Campania ‘Luigi Vanvitelli’—Monaldi Hospital, Piazzale E. Ruggeri, Naples 80126, Italy; Department of Clinical and Experimental Cardiology, Amsterdam UMC, Amsterdam Cardiovascular Sciences, University of Amsterdam, Heart Centre, Meibergdreef 9, Amsterdam 1105AZ, The Netherlands; Division of Geriatric and Intensive Care Medicine, University of Florence and Azienda Ospedaliero Universitaria Careggi, Florence, Italy; Division of Geriatric and Intensive Care Medicine, University of Florence and Azienda Ospedaliero Universitaria Careggi, Florence, Italy; Assistance Publique—Hôpitaux de Marseille, Centre Hospitalier Universitaire La Timone, Service de Cardiologie, France and Aix Marseille Université, C2VN, Marseille 13005, France; Unitat d’Arritmies, Servei de Cardiologia, Hospital Universitari Vall Hebrón i Vall d’Hebron Research Institut, Universitat Autònoma de Barcelona. CIBER-CV, Barcelona, Spain; Department of Cardiology, Ospedali del Tigullio, Lavagna, Italy; Department of Cardiology, Karolinska University Hospital, Stockholm, Sweden; Department of Medicine, Karolinska Institute, Stockholm, Sweden; Division of Geriatric and Intensive Care Medicine, University of Florence and Azienda Ospedaliero Universitaria Careggi, Florence, Italy; Department of Cardiology, IRCCS Istituto Auxologico Italiano, Faint and Fall Research Centre, S. Luca Hospital, Piazzale Brescia 20, Milano 20149, Italy; Department of Medicine and Surgery, University of Milano-Bicocca, Milan, Italy; Department of Cardiology, IRCCS Istituto Auxologico Italiano, Faint and Fall Research Centre, S. Luca Hospital, Piazzale Brescia 20, Milano 20149, Italy

**Keywords:** Syncope, Reflex syncope, Blood pressure, Hypotension, Bradycardia, Ambulatory blood pressure monitoring, Carotid sinus massage, Tilt testing, Standing test

## Abstract

**Background and Aims:**

Identifying the haemodynamic mechanism of autonomic syncope is the essential pre-requisite for effective and personalized therapy aimed at preventing recurrences. The present study assessed the diagnostic efficacy of a two-step assessment.

**Methods:**

Multicentre prospective, cross-sectional, observational study. Patients affected by severe autonomic syncope underwent a two-step assessment including 24-h ambulatory blood pressure monitoring and short cardiovascular autonomic function assessment (SCAFA). SCAFA consisted of carotid sinus massage (CSM), performed in patients ≥40 years old, a passive standing test, and a ‘fast’ head-up tilt test scheduled sequentially during one session on a tilt table.

**Results:**

The study population consisted of 333 patients, 102 ≤ 40 years old and 231 > 40 years old. Any positive response was observed in 298 (89%) patients (92 [92%] in younger and 134 [89%] in older), with hypotensive phenotype accounting for 226 (68%), bradycardic phenotype for 21 (6%) and mixed phenotype for 51 (15%) of cases. The mean duration of the SCAFA procedure was 25 (IQR 20–32) min. Ambulatory blood pressure monitoring, CSM, passive standing, and head-up tilt test were positive in 60%, 15%, 3%, and 71% of patients, respectively. More than one test was positive in 51% and 49% of patients ≤40 and >40 years, respectively. Large inter-centre variability of CSM positivity rate, which remained significant after adjustment for demographic and clinical variables, was observed (*P* = .003).

**Conclusions:**

The standardized 2STEPS protocol offers an easy-to-perform and time-saving diagnostic work-up allowing identification of the haemodynamic mechanism of loss of consciousness in most patients with autonomic syncope. This protocol provides the necessary background for a personalized mechanism-specific therapy.

## Structured Graphical Abstract

**Structured Graphical Abstract:**
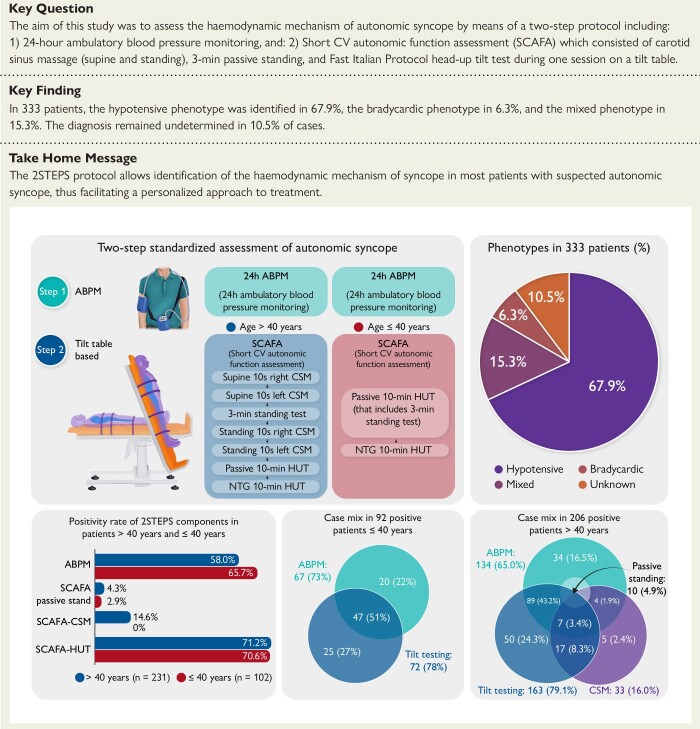
Detection of the haemodynamic mechanism (phenotype) of autonomic syncope by the two-step standardized assessment. ABPM, ambulatory blood pressure monitoring; CSM, carotid sinus massage; CV, cardiovascular; HUT, head-up tilt test; NTG, nitroglycerine; SCAFA, short cardiovascular autonomic function assessment.

**See the editorial comment for this article ‘24-hour ambulatory blood pressure monitoring: a game changer in the management of reflex syncope?’, by P. Mitro, https://doi.org/10.1093/eurheartj/ehae872.**

## Introduction

Different types of transient loss of consciousness events are defined based on their pathophysiological features. The qualifying criterion for syncope is cerebral hypoperfusion that differentiates syncope from non-syncopal forms such as epileptic seizure or psychogenic pseudo syncope.^1^ In patients with a diagnosis of syncope, once a cardiac cause (such as primary intrinsic cardiac arrhythmias or structural flow obstruction) can be ruled out, autonomic neural mechanisms are involved in the genesis of non-cardiac forms.^1–3^ Autonomic (non-cardiac) syncope encompasses both reflex (neurally mediated) syncope and syncope due to orthostatic hypotension as described in the European Society of Cardiology (ESC) guidelines.^1^ Identifying the haemodynamic mechanism of autonomic syncope is the essential pre-requisite for a personalized treatment approach aiming at effectively preventing recurrences. The possible haemodynamic mechanisms underlying autonomic syncope include primary hypotension and asystole/bradycardia of extrinsic cause, corresponding to two different haemodynamic phenotypes, i.e. the hypotensive and bradycardic phenotypes. The choice of therapy—aiming at counteracting hypotension or bradycardia—depends on the detected syncope phenotype identified during the diagnostic work-up.^4^

Autonomic syncope is a common event in the general population and a frequent reason for seeking medical attention, with a consequent impact on high healthcare costs. Moreover, in a significant proportion of patients, syncope might be disabling due to recurrent episodes and/or severe injuries, which may determine activity restriction and poor quality of life.^2,5–7^ In clinical practice, the diagnostic approach to syncope is very heterogeneous, with highly variable adherence to international guideline recommendations. The absence of a systematic approach incurs higher health and social care costs, unnecessary hospitalizations, and diagnostic procedures, while achieving low diagnostic rates and high rates of symptom recurrence. One of the barriers to widespread adherence to guideline protocols is the availability of many diagnostic tests, some of which are time-consuming, expensive, and not fully covered by public healthcare plans.^5–7^

The aim of this study was to assess the diagnostic efficacy of a two-step assessment that consisted of 24-h ambulatory blood pressure monitoring (ABPM) and tilt-table short cardiovascular autonomic function assessment (SCAFA), a set of tests performed in a tilt test laboratory under beat-to-beat haemodynamic monitoring. The study hypothesis was that these two investigations, performed in sequence after the initial evaluation, offer a brief, easy-to-perform, and low-cost diagnostic work-up allowing for the identification of the haemodynamic mechanism of loss of consciousness in most of the patients with autonomic syncope.

## Methods

The 2STEPS study is a multicentre, prospective, cross-sectional, observational study on the effectiveness, and diagnostic efficacy of a two-step standardized approach, including ABPM and tilt table-based SCAFA performed in patients affected by severe autonomic syncope with uncertain mechanisms after the initial evaluation. Eight tertiary diagnostic centres participated in the study. The study recruitment started in April 2023 and ended in March 2024.

### Inclusion criteria

Consecutive patients, >16 years old, affected by severe or recurrent autonomic syncope referred for assessment of the mechanism of syncope after the initial evaluation were recruited. Autonomic syncope was diagnosed when the clinical features were consistent with reflex syncope or orthostatic hypotension, and cardiac syncope was ruled out.^1^ Patients with severe syncope were defined as those who were seeking medical help because affected by recurrent syncopal events impairing quality of life or had syncope with absent or very short prodrome increasing the risk of injuries or were at risk of syncope during high-risk activities (e.g. driving, machine operation, flying, or competitive athletics, etc.).

### Exclusion criteria

1. Non-syncopal causes of real or apparent transient loss of consciousness that may have been incorrectly diagnosed as syncope (e.g. accidental falls, epilepsy, psychogenic pseudo syncope).
2. Established or suspected cardiac syncope in compliance with the criteria of the ESC syncope guidelines.^1^ Specifically, these were the patients with (ⅰ) suspected cardiac arrhythmic syncope [inadequate sinus bradycardia (<50 bpm) or sinoatrial block, second-degree Mobitz I atrioventricular block, second-degree Mobitz II or third-degree atrioventricular block, paroxysmal tachyarrhythmia or ventricular tachycardia, bundle branch block]; (ⅱ) severe structural heart disease and/or significant ECG abnormalities, as defined in *Table 2* of those guidelines.^1^
3. Syncope due to the initial and classical forms of orthostatic hypotension diagnosed at the initial evaluation by typical history and active standing test.
4. Constitutional or drug-induced persistent hypotension was already diagnosed at the initial evaluation by typical history and office or home blood pressure (BP) measurement.
5. Non-severe forms of autonomic syncope, i.e. patients with rare and mild episodes occurring in low-risk situations. In these patients, the investigation of the underlying mechanism of syncope is not necessary, and treatment strategies are mainly based on education on preventive measures, lifestyle modification, and reassurance regarding the benign nature of the condition.^8^

### 2STEPs tests: method and positivity criteria

The order of execution of ABPM and SCAFA was left to the organization of each single centre. The time interval between the two diagnostic components of the 2STEPS protocol was recommended to be <1 month.

#### Ambulatory blood pressure monitoring

Ambulatory blood pressure monitoring was performed using validated oscillometric devices with the most appropriate cuff for arm size (small, medium, or large). Readings were obtained automatically at 15-min intervals between 7 a.m. and 11 p.m. and at 30-min intervals between 11 p.m. and 7 a.m. ABPM recordings were checked for quality, and artefacts were automatically excluded before data collection using the standard criteria incorporated in the analysis software (i.e. pulse pressure >100 mmHg or <20 mmHg, systolic BP (SBP) > 240 mmHg or <50 mmHg, diastolic BP > 140 mmHg or <40 mmHg). Ambulatory blood pressure monitoring recordings including <50 valid measurements and/or <10 valid measurements during the nighttime and/or <70% of expected valid readings were excluded, thus fulfilling the European Society of Hypertension recommendations.^8^ ABPM may help the identification of drug-unrelated persistent hypotension (including constitutional hypotension) or drug-related hypotension, particularly in patients with a white coat effect.^9,10^ ABPM may also reveal hypotensive episodes, i.e. isolated daytime SBP drops.^9,11^ If one of the above findings was detected on ABPM, a hypotensive mechanism of syncope (i.e. hypotensive phenotype) was deemed likely. Drug-unrelated hypotension was defined as 24-h SBP <105 mmHg and daytime SBP <115 mmHg in males and 24-h SBP <98 and daytime SBP <105 mmHg in females, respectively.^12^ Drug-related hypotension was defined as 24-h SBP <120 mmHg in patients receiving BP-lowering medications.^8^ Hypotensive drops were defined as ≥1 episode of daytime SBP <90 mmHg or ≥2 episodes of daytime SBP <100 mmHg^11^

#### Short cardiovascular autonomic function assessment

Short cardiovascular autonomic function assessment consisted of carotid sinus massage (CSM), performed only in patients ≥40 years old, a passive standing test; and a head-up tilt test (HUT) performed sequentially as a single procedure on a tilt table. In the event of BP or heart rate lowering during any of the tests, the following test was started when BP and heart rate had returned to the baseline values or to a stable haemodynamic condition. SCAFA was performed during continuous ECG and beat-to-beat BP monitoring provided by commercially available devices for continuous finger BP monitoring (Finometer®, Finapres Medical Systems, Enchede, The Netherlands, and Task Force® monitor, CNSystem, Graz, Austria), based on the photoplethysmographic volume clamp method.

Carotid sinus massage consisted of manual compression at the site of the maximum carotid pulse, between the angle of the jaw and the cricoid cartilage on the anterior margin of the sternocleidomastoid muscle, with the face rotated contra laterally and posteriorly. The massage was applied firmly up and down on the carotid artery for 10 s on the right, then on the left side in the supine position, and then in the upright position, so that each patient underwent four massages; the time between massages was long enough to allow heart rate and BP values to return to baseline.^1,13^ In the case of induction of syncope during CSM, the following tests (i.e. upright CSM and/or HUT) were performed once BP and heart rate were returned to the baseline values or to a stable haemodynamic condition (*Structured Graphical Abstract*). Positive response was defined as reproduction of spontaneous (pre)syncope, recognized by the patient, with fall in SBP >50 mmHg or below 85 mmHg without an asystolic pause/s >3 s (mixed or vasodepressor form) or with an asystolic pause/s >3 s (cardio inhibitory form).^1,13^ CSM was not performed in patients ≤40 years old.

Passive standing was part of the CSM procedure and was performed for 3 min on the tilt table prior to upright CSM, following supine CSM in all patients >40 years. Positive response was defined as reproduction of spontaneous (pre)syncope in the presence of a fall in SBP ≥20 mmHg and/or standing SBP <90 mmHg within 3 min of standing.^1,13^

HUT was performed according to the ‘fast Italian protocol’.^14^ The fast Italian protocol includes a passive phase of 10 min at an angle of 60–70° followed, in case of negative outcome, by a 10-min nitroglycerine phase consisting of sublingual administration of nitroglycerine spray at a fixed dose of 300–400 µg; the test is continued until complete loss of consciousness occurs (defined by the lack of response to a vocal stimuli and/or loss of muscle tonus and/or jerking movements) or until the completion of the protocol without syncope, whichever occurred first. Compared with the traditional 20 + 15 min protocol, the fast protocol was shown to have a similar positivity rate and similar pre-valence of cardio inhibitory, mixed, and vasodepressor responses.^14^ Therefore, the fast protocol can be used instead of the traditional protocol in clinical practice, allowing for low costs and timesaving. Positive response was defined as reproduction of spontaneous syncope, recognized by the patient, associated with the typical haemodynamic pattern of hypotension and bradycardia without an asystolic pause/s >3 s (mixed or vasodepressor form) or with an asystolic pause/s >3 s (cardioinhibitory form).^1,13^ Pre-syncope episodes were considered a positive response only in the case of delayed orthostatic hypotension.

A summary of the positivity criteria of the 2STEPS tests and their definitions is reported in the Supplementary data online, including diagnostic criteria for hypotensive (Supplementary data online, *Table S1*) and bradycardic (see Supplementary data online, *Table S2*) phenotypes. An example of SCAFA test is shown in the Supplementary data online, *Figure S1*.

### Endpoints

#### Primary endpoint

Diagnostic efficacy: syncope phenotypes

#### Secondary endpoints

Diagnostic efficacy of ABPM and of SCAFA components; case mix of etiologic diagnose; overall time duration of SCAFA procedure

### Statistical analysis

Continuous variables were reported as mean and standard deviation (SD) or median and interquartile range (IQR) in the case of not normally distributed data. Categorical variables were shown as absolute and relative frequencies. Continuous variables were compared using Student’s *t*-test or the non-parametric Mann–Whitney *U* test as appropriate. Comparison of proportions was performed by means of Fisher’s exact test. Multivariable logistic regression analysis was used to assess the contribution of baseline variables to positive responses to CSM, ABPM, and HUT. The automatic stepwise selection of variables was based on the entry criterion of *P* < .1 and the stay criterion of *P* < .05. All tests were two-sided, and *P*-values <.05 were considered statistically significant. Analyses were conducted using MedCalc, version 15.8 (Med Calc Software, Mariakerke, Belgium).

Previous studies showed that 14%–25% of patients with autonomic syncope had a diagnosis of asystole >3 s by CSM and HUT.^2^ Relatively more patients had abnormal SBP drops during ABPM (40%)^11^ and vasodepressor or mixed response by CSM and/or HUT (53%).^15^ We arbitrarily considered 60 patients as the minimum number of patients with bradycardic or mixed phenotype sufficient to provide reliable data. Thus, 300 patients with autonomic syncope would be needed to provide 48–75 patients with asystole >3 s that is 16%–25% of all patients, corresponding to 95% CI.

## Results

The study population consisted of 333 patients, 102 aged ≤40 years and 231 aged >40 years (*Table 1*).

**Table 1:** Characteristics of the study population

	Total population (*n* = 333)	Age ≤40 years (*n* = 102)	Age >40 year (*n* = 231)	*P* value
Mean age, years	54 ± 21	27 ± 7	65 ± 12	-
Mean age at first syncope, years	44 ± 24	19 ± 9	55 ± 21	.001
Males	158 (47)	40 (39)	118 (51)	.06
Syncope, total_number_during_life	4 (2–8)	5 (3–15)	3 (2–6)	.001
Syncope, number_last_year	1 (1–3)	2 (1–3)	1 (1–2)	.03
Syncope, no_prodrome	143 (43)	35 (34)	108 (47)	.04
Syncope, orthostatic trigger	144 (43)	45 (44)	99 (43)	.90
Syncope, severe trauma	36 (11)	4 (4)	32 (14)	.007
Syncope, mild_trauma	135 (36)	38 (37)	97 (42)	.47
Hypotensive medications^a^	129 (39)	4 (4)	125 (54)	.001
Hypotensive medications per patient	1.9 ± 0.9	1.5 ± 1.0	1.9 ± 0.9	.38
Office_mean SBP,_mmHg	128.7 ± 17.7	119.0 ± 10.9	133.0 ± 18.5	.001
ECG, abnormal	42 (13)	4 (4)	38 (16)	.001
Structural heart disease	48 (14)	3 (3)	45 (19)	.001

Values are *n* (%) and continuous variables are given as mean ± SD or median (interquartile range) as appropriate.

^a^Hypotensive medications included antihypertensive drugs and psychoactive drugs with known hypotensive effects.^2^

### Endpoints

The results for the primary and secondary endpoints are shown in the *Table 2*. Any positive response of the 2STEPS protocol was observed in 298 (89%) patients, with the hypotensive phenotype accounting for 226 (68%) of cases, the bradycardic phenotype for 21 (6%) of cases, and the mixed phenotype for 51 (15%) of cases. The diagnosis remained unknown in 11%. The total positivity rate was similar in younger and older patients. An asystolic reflex was present in 23 (23%) and 49 (21%) of patients ≤40 and >40 years, respectively. The SCAFA procedure had a mean duration of 25 (IQR 20–32) min.

**Table 2:** Results: diagnostic efficacy of the 2STEPS protocol and its components, by age groups

	Total population (*n* = 333)	Age ≤40 years (*n* = 102)	Age >40 years (*n* = 231)	*P* value
**Primary endpoint**				
Any positive response	298 (89)	92 (90)	206 (89)	.84
Hypotensive phenotype	226 (68)	69 (68)	157 (68)	1.00
Bradycardic phenotype	21 (6)	12 (12)	9 (4)	.01
Mixed phenotype	51 (15)	11 (11)	40 (17)	.14
All negative responses	35 (11)	10 (10)	25 (11)	.85
**Secondary endpoints**				
ABPM total positive responses	201 (60)	67 (66)	134 (58)	.22
Drug-unrelated persistent hypotension	33 (10)	17 (17)	16 (7)	.009
Drug-related persistent hypotension	50 (15)	0 (0)	50 (22)	<.001
SBP drops, total	182 (55)	66 (65)	116 (50)	.14
SBP drops alone	118 (35)	50 (49)	68 (29)	.008
SCAFA total positive responses	246 (74)	73 (72)	173 (75)	.59
CSM positive responses^a^	33/226 (15)	—	33/226 (15)	
Mixed forms	8/226 (3)	—	8/226 (3)	
Cardioinhibitory form	25/226 (11)	—	25/226 (11)	
Passive standing test	10 (3)	—	10 (4)	
Tilt testing positive responses^b^	235/331 (71)	72 (71)	163/229 (71)	1.00
Mixed/Vasodepressor form	168/331(51)	47 (46)	121/229 (53)	9.28
Cardioinhibitory form	51/331 (15)	23 (23)	28/229 (12)	.02
Delayed orthostatic hypotension	16 (5)	2 (2)	14/229 (6)	.16
SCAFA total time, min	27.6 ± 10.8	25.2 ± 9.4	27.8 ± 8.3	.014

Abbreviations: ABPM, ambulatory blood pressure monitoring; CSM, carotid sinus massage; SBP, systolic blood pressure; SCAFA, short cardiovascular autonomic function assessment.

Values are *n* (%) and continuous variables are given as mean ± SD.

^a^CSM was not performed in patients ≤40 years and in 5 patients >40 years who had a history previous TIA/stroke.

^b^Tilt testing was not performed in two patients who refused the test.

**Figure 1:**
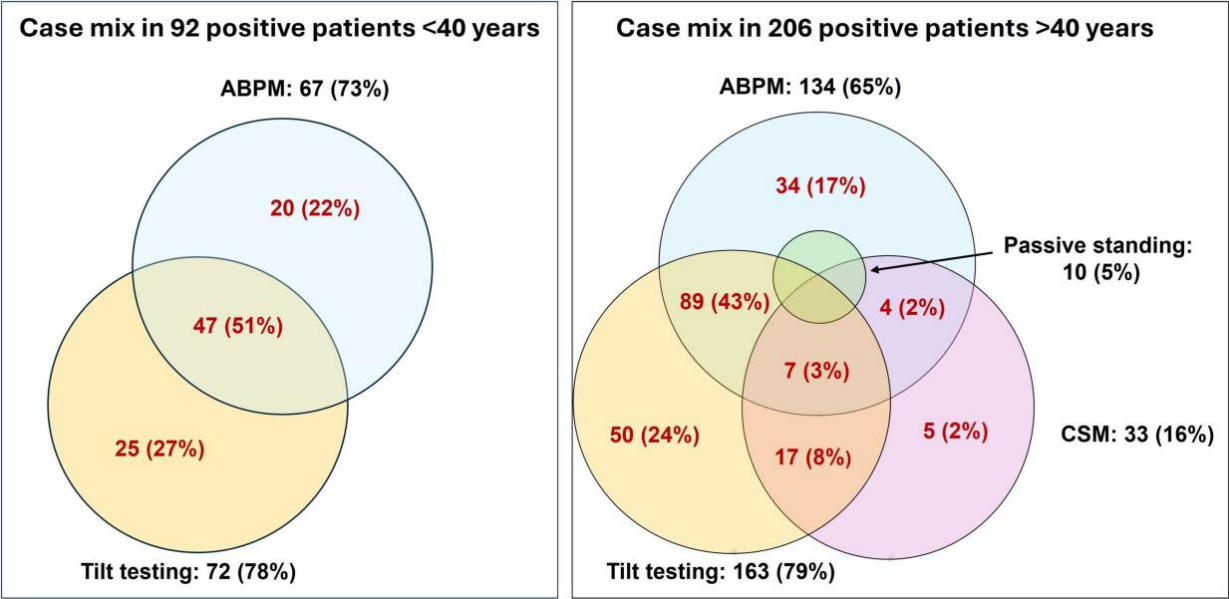
Case mix of positive responses in patients ≤40 years and in those >40 years of age. Proportions refer to the total number of patients with a positive response in each age subgroup. Carotid sinus massage means carotid sinus massage. In patients >40 years, passive standing was positive in four patients with positive carotid sinus massage, 7 patients with positive tilt testing, and in six patients with positive ABPM; in 6 out of 10 cases, 3 or 4 tests were positive in the same patient. Passive standing was never positive alone

All tests, alone or in association with other tests, contributed to the diagnostic efficacy. The case mix of positive responses is shown in *Figure 1*. More than one test was positive in 47 (51%) of 92 positive patients ≤40 years and in 100 (49%) of 206 positive patients >40 years. While ABPM and passive standing contributed to the diagnosis of hypotensive forms, CSM and HUT contributed to the diagnosis of both hypotensive and bradycardic forms. A mixed phenotype was diagnosed when two or three tests were positive in the same patient and showed contrasting results. ABPM revealed a hypotensive susceptibility in 37 patients (11%) who had an asystolic response with CSM or HUT. Even if no patient achieved a diagnosis only based on the passive standing test, the latter provided a diagnosis of mixed phenotype in four patients who had an asystolic response to CSM or HUT. Among 33 patients with positive CSM, 11 had a diagnosis while supine (7 on the right side, 2 in the left side, and 2 in both sides) and 28 had a diagnosis while standing (18 on the right side, 7 on the left side, and 3 on both side). In five patients, CSM was positive only in the supine position, while six patients showed a positive response to both supine and standing CSM; among the latter, two patients showed a cardioinhibitory positive response while supine and a vasodepressor positive response while standing. HUT was positive during the passive phase in 20 cases (9%) and during the active phase in 215 cases (91%).

In multivariable analysis, older age (odds ratio of 1.05 per increasing year, 95% CI 1.01–1.10) and male sex (odds ratio of 4.0 [95% CI 1.59–10.1]) were predictors of a positive response to CSM. Presence of prodromes (odds ratio of 1.59 [95% CI 1.04–2.38]) and lower values of office SBP (odds ratio of 1.02 per 1 mmHg decrease [95% CI 1.01–1.04]) were predictors of positive response to ABPM. No clinical variable predicted a positive response to tilt testing.

### Inter-centre variability of carotid sinus massage

**Figure 2:**
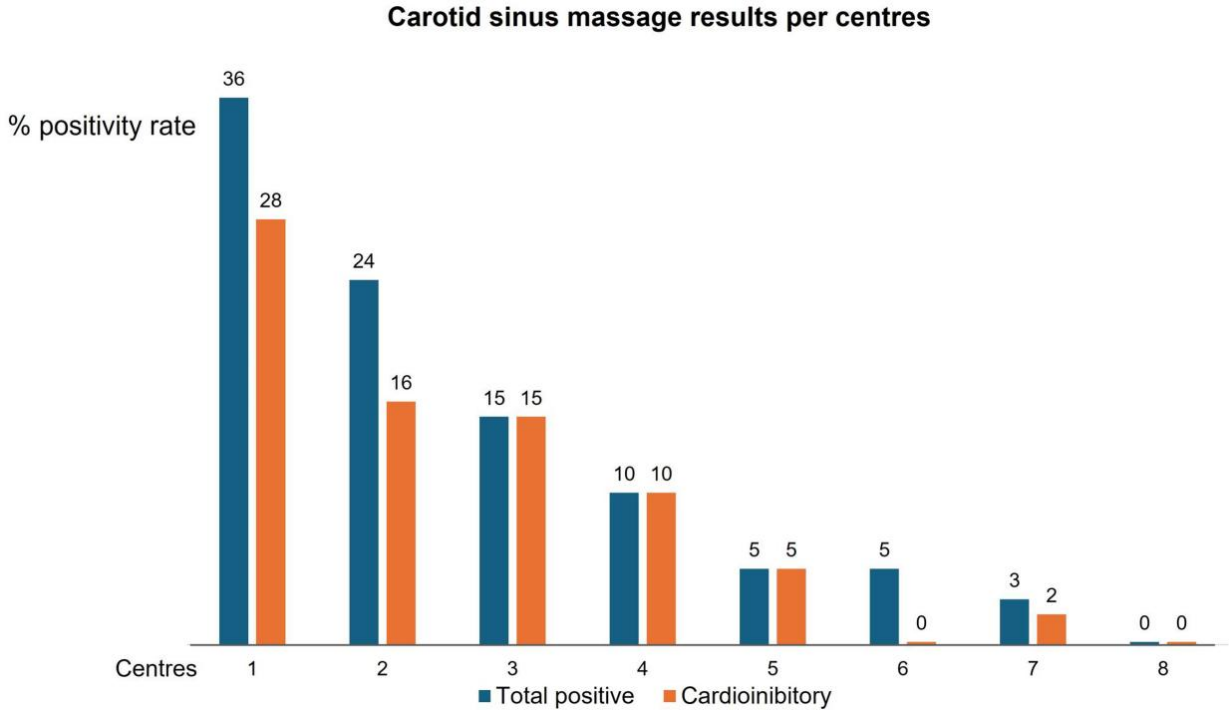
Positivity rate of carotid sinus massage in eight centres (total 226 patients >40 years)

While the positivity rates of ABPM and HUT were sufficiently homogeneous between centres, the results of CSM showed a great heterogeneity (*Figure 2*). In the four centres with the highest positivity rate (*n* = 118 patients), CSM was positive in 30 patients (25%) and a cardio inhibitory response was observed in 23 (19%) of them. Conversely, in the other four centres with the lowest positivity rate (*n* = 113 patients), CSM was positive in only four patients (3%) and a cardioinhibitory response was observed in 2 (2%) of them (*P* = .0001). The difference remained highly significant after adjustment for the other clinical variables listed in the Supplementary data online, *Table S3*, with an odds ratio of 9.8 (95% CI 2.8–34; *P* = .003).

## Discussion

**Figure 3:**
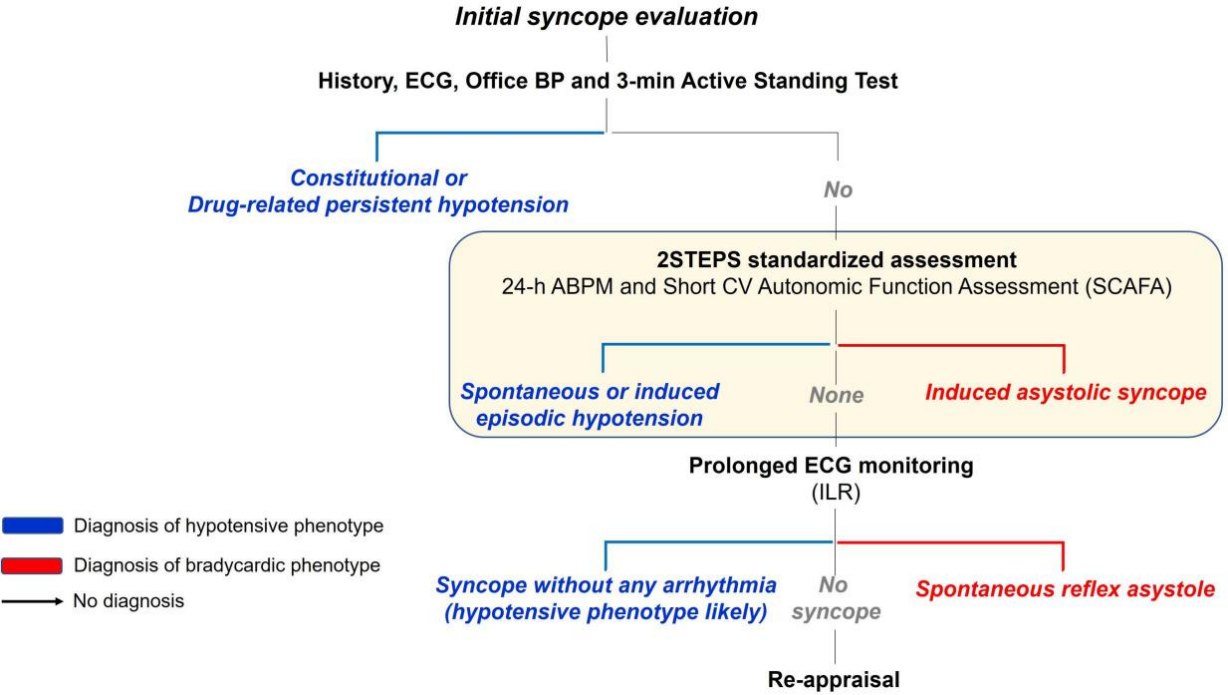
Diagnostic work-up of autonomic syncope by mechanism. The initial evaluation consists of history taking, standard 12-lead ECG, and automated blood pressure measurement supine and during 3 min of active standing. During the initial evaluation, persistent hypotension (either constitutional or drug-related) and the initial and classical forms of orthostatic hypotension can be identified, and cardiac or non-syncopal causes of transient loss of consciousness are excluded from further assessment. When the results of the initial evaluation are unremarkable, the second phase consists of 24 h ambulatory blood pressure monitoring and Short Cardiovascular Autonomic Function Assessment performed during continuous ECG and BP monitoring as described in this study. Finally, an implantable loop recorder is usually needed if uncertainty persists on the mechanism of syncope. Modified with permission from Brignole et al.^4^

This study investigated the diagnostic efficacy of a two-step assessment, including ABPM and SCAFA, performed after the initial evaluation in patients with severe syncope, once cardiac causes have been excluded. The study showed that two tests, i.e. ABPM and SCAFA, were able to determine the likely mechanism of autonomic syncope in 89% of cases with similar diagnostic efficacy in patients ≤40 years and in those >40 years of age. All 2STEPS components contributed and were necessary for a comprehensive assessment of the possible haemodynamic phenomena underlying loss of consciousness. ABPM is a widely available, low-cost, and easy-to-use diagnostic tool, which has recently emerged as a valuable instrument for the investigation of syncope with hypotensive phenotype.^11^ SCAFA combines three well-known diagnostic tests in the same procedure, with a mean duration of 25 (IQR 20–32) min. This battery thus appears to be time- and cost-saving and more suitable to the time constraints of routine clinical practices. Moreover, the high diagnostic efficacy of 2STEPS prevents overutilization of tests that are often indiscriminately ordered for the diagnosis of syncope.^2,5–7,16^ Finally, in the 2STEPS, protocol, the definitions of positive responses (see also Supplementary data online, *Tables S1* and *S2*) are standardized, based on the best available evidence, making interpretation of results and the choice of subsequent mechanism-based therapy uniform among physicians and easy to be applied to their patients. Given these premises, the 2STEPS protocol is expected to have a substantial, positive impact on the work-up of patients with autonomic syncope when the results of the initial evaluation are unremarkable or inconclusive and before an implantable loop recorder (ILR) is considered (*Figure 3*).

The overall pre-valence of CSS was probably underestimated in the present study. The pre-valence of CSS showed large inter-centre variability in our study (*Figure 2*), reaching 25% in the four centres with higher positivity rates. Since the centres had similar clinical characteristics of patients at enrollment (see Supplementary data online, *Table S3*), we can suppose that the difference was mainly due to inter-operator variability. Despite CSM being an old test routinely performed over the last 100 years, the method of performing CSM is still largely operator-dependent with consistent interoperator variability. Only during the last decade, the role of CSM has been better defined, and the test has been placed in an appropriate environment (i.e. the tilt test laboratory) and is recommended to be performed under obligatory continuous BP monitoring for accurate detection of the vasodepressor/hypotensive component. In the absence of continuous BP monitoring, rapid changes in BP caused by inappropriate baroreceptor response will not be detected, and the syncope mechanism will remain unexplained. CSM is also an important component of 2STEPS because it allows detection of cardio inhibitory forms that otherwise would be missed.^15^ CSM is largely underused in clinical practice outside syncope facilities, especially in the USA, with a pre-valence ranging from 0.07% to 8%.^17–20^ Thus, there is an urgent need for the scientific community to define the most appropriate method of CSM implementation and to start educational programmes to help physicians to perform this procedure properly.

The combination of ABPM and SCAFA is original and has never been assessed previously. The diagnostic efficacy of the 2STEPS protocol was higher than that reported in the literature with other diagnostic approaches. For example, the diagnostic efficacy of a comprehensive diagnostic evaluation (including multiple tests) in two large populations referred to the syncope unit was 79% in Sweden^21^ and 82% in Italy.^7^ ABPM alone was diagnostic in 54 patients (16%) in whom the diagnosis would have remained unexplained in the absence of this test. ABPM revealed a hypotensive susceptibility in 37 patients (11.1%) who had an asystolic response with CSM or HUT, thus allowing in these patients the diagnosis of mixed phenotype that otherwise would have been missed. This finding has relevant clinical implications because in these patients, dual therapy against both hypotension and bradycardia is probably required to prevent syncopal recurrences.^4^ Finally, ABPM revealed SBP drops in 182 patients (55%). Therapy aimed at abolishing such drops (hypotensive drug deprescribing or pre-scribing of drugs that increase BP) has been suggested as a target for preventing syncopal recurrences.^22^

The combination of CSM and HUT revealed a similar proportion of asystolic reflex in patients ≤40 years and in those >40 years, even if in the latter it was more frequently associated with hypotensive reflex. The lower rate of asystolic HUT observed in older patients was balanced by a higher rate of asystolic responses to CSM. This finding confirms that the relative proportion rate of asystolic syncope is not influenced by age and is consistent with the findings of previous studies with HUT^23,24^ and with implanted loop recorders.^25^

In the minority of patients in whom the diagnosis remains unexplained after the 2STEPS protocol, further investigations are generally indicated. Among these, long-term monitoring with ILR should be considered as the first-line option, either to reveal asystolic reflex syncope not recognized by CSM and HUT or to document unrecognized primary cardiac arrhythmias, especially in patients with underlying structural heart disease or other high-risk features.^26,27^

In the study population, there was a high prevalence of hypotensive phenotype. This can be explained by the high proportion of patients with persistent hypotension (constitutional hypotension) and patients with drug-related persistent hypotension diagnosed by ABPM, which accounted for a quarter of patients. Such patients were usually excluded from other studies in the literature. However, our findings are more representative of the general population of patients referred to a syncope unit because they were affected by severe forms of autonomic syncope and are consistent with the general knowledge of the pre-vailing hypotensive mechanism of autonomic syncope.

### Limitations

Some limitations of the present study should be acknowledged. First, the diagnosis of autonomic syncope—and the rule-out of established/suspected cardiac syncope—was made after the initial evaluation. This means that the diagnosis is most often pre-sumptive, as typically occurs in this field. We cannot completely exclude that some patients, who were mistakenly assigned to the autonomic group, could have a cardiac form. Second, the order of execution of ABPM and SCAFA was decided by each participating centre, and the temporal range of their execution was not reported. The initial and classical forms of orthostatic hypotension were not formally assessed in this study during SCAFA. In particular, SCAFA is unable to investigate initial orthostatic hypotension which is only associated with active standing. As beat-to-beat BP monitoring may not be available during the initial evaluation in some centres, we cannot exclude that this protocol might lead to under diagnosis of initial orthostatic hypotension. However, in our study, the initial 3-min passive standing on the tilt table was able to detect 10 cases of symptomatic fall in BP that could resemble the initial and classical orthostatic hypotension, detected by active standing. We acknowledge the lack of a beat-to-beat monitored active standing test in the proposed autonomic assessment protocol, which should be considered in future recommendations. Finally, in the event of BP or heart rate lowering during any of the tests, the following test was started when BP and heart rate had returned to the baseline values or to a stable haemodynamic condition, leaving to the physician’s judgment the decision of stability rather than using a fixed cautionary standard period (e.g. 5 min). This lack of standardization might have affected the diagnostic efficacy of SCAFA.

### Practical implications

Identifying the precise haemodynamic mechanism of autonomic syncope is the essential pre-requisite for a personalized approach aimed at guiding the subsequent treatment. The treatment outcome of the patients was not the aim of the present study. We anticipate that a prospective ongoing study will assess the long-term results of the mechanism-based therapy of the patients with hypotensive phenotype (ClincalTrials.gov identifier: NCT06513650). However, based on the current state of the art,^4^ cardiac pacing, cardioneuroablation, and adenosine-receptor antagonist drugs (such as theophylline), are proven effective therapies for patients with a dominant bradycardic phenotype of extrinsic cause (definition provided in the Supplementary data online, *Table S2*). Conversely, in patients with a dominant hypotensive phenotype, the therapy of choice should be either discontinuation of BP–lowering drugs and/or psychoactive drugs in patients with drug-related persistent or intermittent hypotension. In patients with drug-unrelated persistent or intermittent hypotension (definition provided in the Supplementary data online, *Table S1*), administration of BP-elevating agents (such as fludrocortisone and midodrine) is recommended, supported by non-pharmacological methods such as the use of elastic compression garments. An important finding of the present study is the identification of patients with mixed phenotype in whom a combination of the above therapies is usually necessary to prevent syncopal recurrences.

## Conclusion

The 2STEPS diagnostic protocol, consisting of 24-h ABPM and CV autonomic tests, is easy to apply, time-saving, and highly efficient in providing a rationale for mechanism-based therapy in 89% of patients affected by autonomic syncope. For all the above reasons, we propose that the 2STEPS protocol should be considered as a new standard for clinical practice in syncope management.

## Supplementary data

Supplementary data are available at *European Heart Journal* online.

## Supplementary Material

ehae907_Supplementary_Data

## Declarations

### Data Availability

The datasets generated during and/or analyzed during the current study are available upon reasonable request.

### Pre-registered Clinical Trial Number

ClincalTrials.gov identifier: NCT05728255.
