# Supplementary material for: Mechanism of syncope: role of ambulatory blood pressure monitoring and cardiovascular autonomic function assessment
Source: Eur Heart J. 2024 Dec 30;46(9):827–35. doi: 10.1093/eurheartj/ehae907 (PMC11879164; doi:10.1093/eurheartj/ehae907)

**Supplementary documents**

**Test for autonomic syncope**

Definitions of positive responses of the tests referred to the diagnostic work-up shown in the Figure 4.

**Table S1. Hypotensive phenotype: diagnostic criteria.**

| **Diagnosis** | **Definition** | **Test** | **Blood pressure cut-offs** |
| --- | --- | --- | --- |
| Drug-unrelated persistent hypotension (constitutional hypotension) | Persistently low BP in the absence of hypotensive medications | 24-hour ABPM (1) | *Males*  24 h SBP <105 mm Hg  Daytime SBP <115 mm Hg  *Females*  24h SBP <98 mm Hg  Daytime SBP <105 mm Hg |
| Drug-related persistent hypotension | SBP values persistently below the recommended target in patients receiving hypotensive medications | 24-hour ABPM (2) | 24h SBP <120 mmHg |
| Hypotensive (intermittent) episodes | Orthostatic hypotension (classical form) | Active and passive standing | Symptomatic SBP fall ≥20mmHg or standing SBP <90mmHg within 3 min of active standing during the initial evaluation or during passive standing while performing SCAFA |
|  | Orthostatic hypotension  (initial form) | Active standing | Initial OH is characterized by a BP decrease on standing of >40 mmHg for systolic BP and/or >20 mmHg for diastolic BP within 15 s of standing. BP then spontaneously and rapidly returns to normal, so the period of hypotension and symptoms is short (<40 s) but may still cause syncope. A beat-to-beat BP monitoring is preferable. |
|  | Hypotensive drops | 24-hour ABPM (3) | ≥1 episodes of daytime SBP <90 mmHg  ≥2 episodes of daytime SBP <100 mmHg |
| Hypotensive reflex syncope | 1) Induction of syncope during tilt table test | Tilt table test (4) | Typical haemodynamic pattern of mixed or vasodepressor vasovagal syncope with hypotension and bradycardia but without asystolic pauses >3 s.  Delayed orthostatic hypotension: prolonged (>3 min) hypotensive prodromes that may be followed by reflex syncope in absence of fall in heart rate |
|  | 2) Reproduction of (pre)syncope during carotid sinus massage (method of symptoms) | Carotid sinus massage | Reproduction of spontaneous (pre)syncope, recognized by the patient itself, with fall in SBP >50 mmHg or below 85 mmHg and absence of asystolic pause/s >3 s |

ABPM, ambulatory blood pressure monitoring; BP, blood pressure; DBP, diastolic blood pressure; SBP, systolic blood pressure

**Table S2. Bradycardic phenotype: diagnostic criteria**

| **Diagnosis** | **Definition** | **Test** | **CI cut-offs** |
| --- | --- | --- | --- |
| CI reflex syncope | 1)Reproduction of spontaneous symptoms during CSM (method of symptoms) | Supine and standing CSM | Reproduction of spontaneous (pre)syncope, recognized by the patient itself, with fall in SBP >50 mmHg and asystolic pause/s >3 s. |
|  | 2)Reproduction of spontaneous syncope during tilt table test | Tilt table test (4) | Typical ECG pattern of vasovagal syncope during hypotension and asystolic pause >3 s) |
|  | 3)Asystolic pauses of likely reflex origin during prolonged ECG monitoring (ILR) | Prolonged ECG monitoring (ILR) | Typical ECG pattern of asystolic (>3 s) vasovagal syncope or documentation of asymptomatic asystolic pause >6 s of likely reflex origin |

CI = cardioinhibitory; CSM = carotid sinus massage; SBP= systolic blood pressure; ILR = implantable loop recorder

**References of Table S1 and S2**

When not cited specifically, the reported definitions in the Tables S1 and S2 is the 2018 ESC guidelines on Syncope:

- Brignole M, Moya A, De Lange FJ, Deharo J, Elliott PM, Fanciulli A, et al. 2018 ESC Guidelines for the diagnosis and management of syncope. European Heart Journal. 2018; 39:1883-1948

- Brignole M, Moya A, de Lange FJ, Deharo J, Elliott PM, Fanciulli A, et al. Practical Instructions for the 2018 ESC Guidelines for the diagnosis and management of syncope. Eur Heart J. 2018; 39: e43-80.

Specific references, other than the above:

1. Owens PE, Lyons SP, O'Brien ET. Arterial hypotension: prevalence of low blood pressure in the general population using ambulatory blood pressure monitoring. J Hum Hypertens. 2000; 14: 243-247.

2. Stergiou GS, Palatini P, Parati G, O'Brien E, Januszewicz A, Lurbe E, et al. 2021 European Society of Hypertension practice guidelines for office and out-of-office blood pressure measurement. J Hypertens. 2021; 39: 1293-1302.

3. Rivasi G, Groppelli A, Brignole M, Soranna D, Zambon A, Bilo G, et al. Association between hypotension during 24 h ambulatory blood pressure monitoring and reflex syncope: the SynABPM 1 study. Eur Heart J. 2022; 43: 3765-3776.

4. Russo V, Parente E, Tomaino M, Comune A, Sabatini A, Laezza N, et al. Short-duration head-up tilt test potentiated with sublingual nitroglycerin in suspected vasovagal syncope: the Fast Italian Protocol. European Heart Journal. 2023; 44: 2473-2479

**Table S3-** Carotid sinus massage: comparison between the 4 centres with the highest positivity rate versus the 4 centres with the lowest positivity rate in 231 patients >40 years.

|  | **High positivity**  **(n=118)** | **Low positivity (n=113)** | **P value** |
| --- | --- | --- | --- |
| Total positive CSM | 30 (25.4) | 4 (3.4%) | 0.0001 |
| Positive cardioinhibitory form | 23 (19.5) | 2 (1.7%) | 0.0001 |
| Mean age, yrs | 66.5±12.1 | 64.0±13.3 | 0.12 |
| Mean age at first syncope, yrs | 54.6±21.8 | 54.9±19.5 | 0.90 |
| Males | 60 (50.8) | 58 (51.3) | 1.00 |
| Syncope, total_number_during_life | 3 (2-6) | 3 (2-6) | 0.23 |
| Syncope, number_last_year | 1 (1-2) | 1 (1-2) | 0.79 |
| Syncope, no_prodrome | 64 (54.2) | 44 (38.9) | 0.69 |
| Syncope, orthostatic trigger | 54 (45.8) | 45 (39.8) | 0.43 |
| Syncope, severe_trauma | 15 (12.7) | 17 (15.0) | 0.70 |
| Syncope, mild_trauma | 43 (36.4) | 54 (47.8) | 0.08 |
| Hypotensive medications | 60 (50.8) | 65 (57.5) | 0.36 |
| Office_mean SBP_mmHg | 133.6±18.0 | 132.5±19.0 | 0.65 |
| ECG, abnormal | 21 (17.8) | 17 (15.0) | 0.60 |
| Structural heart disease | 15 (12.7) | 30 (25.4) | 0.01 |

CSM = carotid sinus massage; SBP = systolic blood pressure

**Figure S1. SCAFA test.**

The figure shows an example of a SCAFA test performed on a tilt table during continuous monitoring of heart rate (ECG) and systolic, mean, and diastolic blood pressure (photopletismographic method). The top panel shows the heart rate trend. The botton panel shows blood pressure trend. The sequence of tests (red vertical lines) was: 1) Supine, rest; 2) Supine, right carotid sinus massage (CSM); 3) Supine, left CSM; 4) Passive standing test; 5) Standing right CSM; 6) Standing, left CSM; 7) Head-up tilt test (HUT), passive phase; 8) HUT, nitroglycerine phase; 9) tilt-down

The supine right and left CSM caused hypotension and bradycardia without reproduction of spontaneous symptoms. The standing right CSM caused syncope recognized by the patient. The nitroglycerine HUT phase ended with a mixed form of vasovagal syncope with hypotension and bradycardia without asystole >3 s.


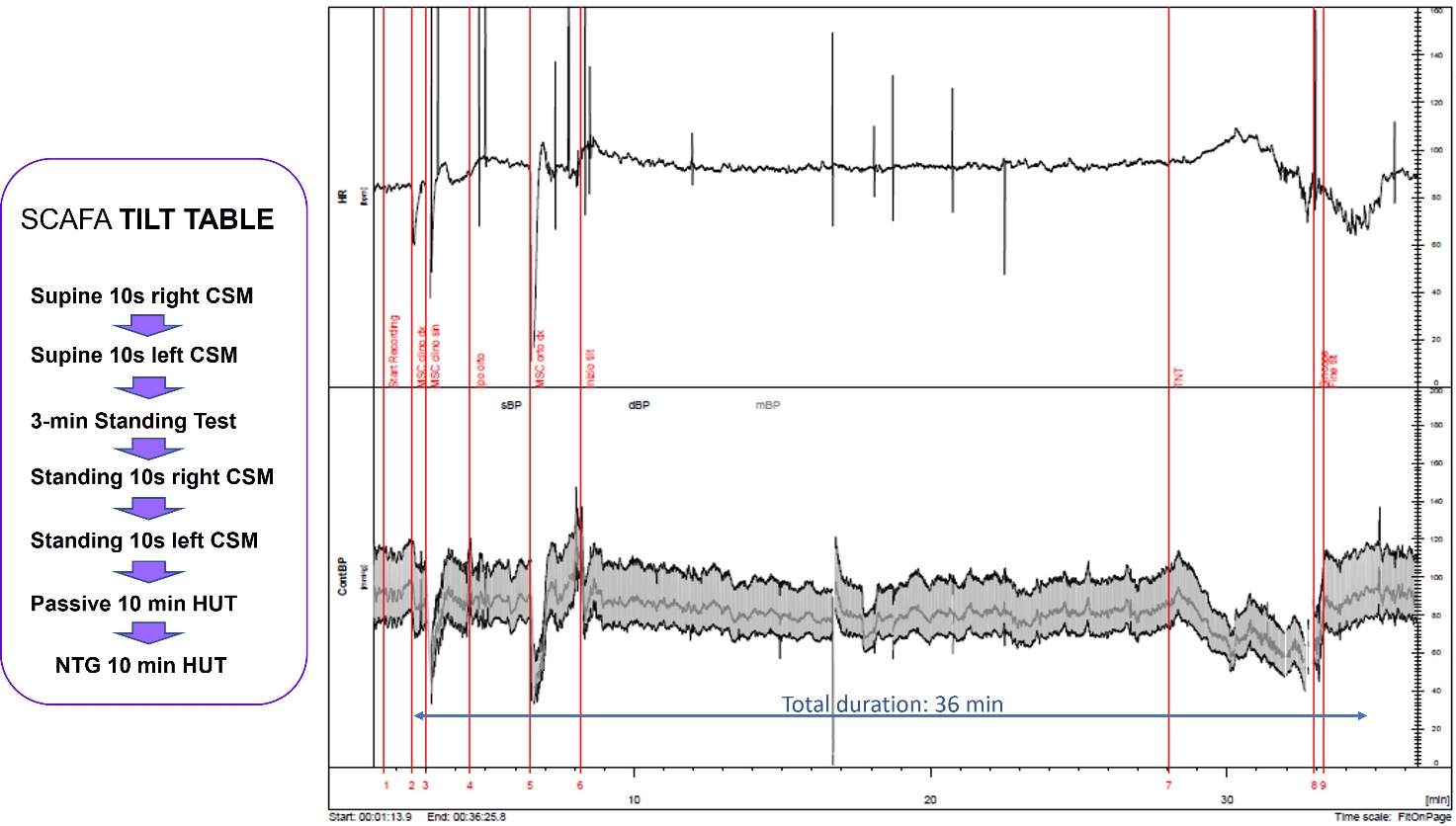

Supplement: ehae907_Supplementary_Data [file ehae907_supplementary_data.docx]
